# Metabolism of BYZX in Human Liver Microsomes and Cytosol: Identification of the Metabolites and Metabolic Pathways of BYZX

**DOI:** 10.1371/journal.pone.0059882

**Published:** 2013-03-29

**Authors:** Lushan Yu, Yan Jiang, Lu Wang, Rong Sheng, Yongzhou Hu, Su Zeng

**Affiliations:** 1 Laboratory of Pharmaceutical Analysis and Drug Metabolism, College of Pharmaceutical Sciences, Zhejiang University, Hangzhou, China; 2 Zhejiang University-Ecole Normole Superienre Joint Laboratory of Medicinal Chemistry, College of Pharmaceutical Sciences, Zhejiang University, Hangzhou, China; University of Ulster, United Kingdom

## Abstract

BYZX, [(E)-2-(4-((diethylamino)methyl)benzylidene)-5,6-dimethoxy-2,3-dihydroinden-one], belongs to a series of novel acetylcholinesterase inhibitors and has been synthesized as a new chemical entity for the treatment of Alzheimer’s disease symptoms. When incubated with human liver microsomes (HLMs), BYZX was rapidly transformed into its metabolites M1, M2, and M3. The chemical structures of these metabolites were identified using liquid chromatography tandem mass spectrometry and nuclear magnetic resonance, which indicated that M1 was an *N*-desethylated and C = C hydrogenation metabolite of BYZX. M2 and M3 were 2 precursor metabolites, which resulted from the hydrogenation and desethylation of BYZX, respectively. Further studies with chemical inhibitors and human recombinant cytochrome P450s (CYPs), and correlation studies were performed. The results indicated that the *N*-desethylation of BYZX and M2 was mediated by CYP3A4 and CYP2C8. The reduced form of β-nicotinamide adenine dinucleotide 2′-phosphate was involved in the hydrogenation of BYZX and M3, and this reaction occurred in the HLMs and in the human liver cytosol. The hydrogenation reaction was not inhibited by any chemical inhibitors of CYPs, but it was significantly inhibited by some substrates of α,β-ketoalkene C = C reductases and their inhibitors such as benzylideneacetone, dicoumarol, and indomethacin. Our results suggest that α,β-ketoalkene C = C reductases may play a role in the hydrogenation reaction, but this issue requires further clarification.

## Introduction

BYZX [(E)-2-(4-((diethylamino)methyl)benzylidene)-5,6-dimethoxy-2,3-dihydroinden-one] was one of the synthetic acetylcholinesterase (AChE) inhibitors selected for the treatment of Alzheimer’s disease symptoms [1]. BYZX belongs to 5,6-dimethoxy-indan-1-one derivatives designed from donepezil and is similar to donepezil, fits well into the active-site gorge of AChE, and simultaneously binds to the central subsite and the peripheral anionic site [2]. BYZX can significantly improve chemical-induced learning and memory impairments in rodents and protect PC12 cells from apoptosis induced by hydrogen peroxide [3]. Currently, BYZX is being developed as a potential candidate for therapeutic intervention in neurodegenerative diseases.

The disposition profile of a drug plays a critical role in its efficacy and side effects. Drugs with a rapid metabolic clearance are likely to have a high degree of inter- and intrapatient variability [4]. Moreover, the occurrence of reactive drug metabolites is the major cause of drug-induced toxicities [5]–[7]. On the other hand, metabolites may have stronger bioactivities than its precursor and thus may be novel potential therapeutic candidates, or may explain the reason of drug potency. Therefore, increasing attention has been paid to metabolite formation and metabolic pathways, and drug metabolism research now spans the continuum throughout drug development [8].

We have reported the results of a preliminary metabolic study of BYZX in the human liver microsomes (HLMs) in which only the *N*-desethyl metabolite was detected [9]. However, these results were not sufficiently comprehensive because of the low resolution of the metabolite in chromatographic separation. In this study, we identified 2 other metabolites of BYZX. Cytochrome P450 2C8 (CYP2C8) and CYP3A4 were involved in the *N*-desethylation of BYZX. Interestingly, C = C reduction reaction, an unusual reaction in drug metabolism mediated by microsomes, was found in the BYZX metabolism, which also happened in the human liver cytosol.

## Materials and Methods

### Materials and Chemicals

BYZX, metabolite 2 (M2) and BYYT-25 (internal standard) were offered by Department of Medicinal Chemistry, Zhejiang University (Hangzhou, China); Trisodium isocitric acid, isocitric dehydrogenase, Nicotinamide adenine dinucleotide phosphate sodium salt (β-NADP) and its reduced form (β-NADPH), 1-aminobenzotriazole, α-naphthoflavone, ketoconazole, quercetin, sulfaphenazole, ticlopidine, indomethacin and finasteride, and specific substrates of CYPs as well as their metabolites (listed in Section 2.8.2) were purchased from Sigma-Aldrich (St. Louis, MO, USA); quinidine and sodium diethyldithiocarbamate were purchased from the Chemical Reagent Factory of ShangHai (Shanghai, China); sertraline was gained from Shanghai Institute of Pharmaceutical Industry (Shanghai, China); benzalacetone was purchased from Aladdin Reagent Corporation (Shanghai, China); dicoumarol was purchased from J&K Scientific Ltd. (Shanghai, China). All other chemicals of analytical or HPLC grade were purchased from standard commercial sources.

Commercial baculovirus-insect cell-expressed human cytochrome P450 enzymes (CYPs) (supersomes) including CYP1A2, 2A6, 2B6, 2C8, 2C9, 2C19, 2D6, 2E1 and 3A4 were purchased from BD Gentest (Woburn, MA). The individual human liver microsomes (HLMs), pooled HLMs and human liver cytosol were obtained from Research Institute for Liver Disease (Shanghai, China) and were stored at −80°C; male Sprague Dawley rats (180±20 g) were purchased from Zhejiang University Laboratory Animal Center and were kept in a breeding room with normal foods and water before use.

### Incubation of BYZX with the Pooled HLMs

BYZX (20 µM) was incubated in a 200 µL of reaction buffer solution containing 0.1 M Tris-HCl (pH 7.4), 0.15 M MgCl_2_, NADPH-generating system (9.7 mM dl-isocitrate trisodium and 0.07 unit of isocitrate dehydrogenase), and 1 mg/mL pooled HLMs. The incubation mixture was preincubated at 37°C for 5 min, and then the reaction was initiated by adding 4 µL of NADP/NADPH solution (0.026 M NADP and 0.012 M NADPH in 1% NaHCO_3_ solution). After incubation at 37°C for 120 min, 200 µL of cold methanol was added to terminate the reaction and precipitate the protein. The mixture was vortexed for 1 min and centrifuged at 13 000 rpm for 15 min. 7 µL of the supernatant was injected into the LC system.

### Qualitative Analysis of Metabolite in the HLMs by Liquid Chromatography Mass Spectrometry (LC-MS)

BYZX and its metabolites in the HLMs incubation were detected by an Acquity UPLC-TQD system (Waters Acquity, Waters, Milford, MA) equipped with an electro-spray interface (ESI) operated in triple quadrupole mode (TQD, Waters, USA). Chromatographic separation was carried out on an Agilent ZORBAX SB-C_18_ column (2.1 mm×50 mm, 1.8 µm, Agilent) with a binary gradient system consisting of 0.1% formic acid in water and methanol. The gradient started at 15% methanol and then raised up linearly to 50% over 8.0 min, followed by a further increase to 95% in 1.0 min and maintained for 1.0 min to flush the column, rapidly returned to 15% and equilibrated for 1.0 min. The column temperature was 40°C, and the flow rate was 0.3 mL/min. The ESI source of TQD was set as follow: capillary voltage, +3.5 kV; cone voltage, 30 V; extractor voltage, 3 V; source temperature, 120°C; desolvation temperature 350°C; desolvation gas flow, 500 L/Hr; the detection was performed under the full scan from *m/z* 150 to *m/z* 600; the fragment ions of BYZX and each of its metabolites were acquired at a collision voltage of 40 eV with a collision gas flow of 0.12 mL/min, and the scan range was from *m/z* 50 to *m/z* 375.

The accurate molecular weights of the metabolites were acquired by using an Agilent 6530 RRLC-Q-TOF system. The chromatographic column and mobile phase gradient program were the same as the aforementioned method. The ESI source was set as follow: source temperature, 325°C; drying gas flow rate, 10 L/min; sheath gas temperature, 350°C; sheath gas flow rate, 10 L/min; capillary voltage, +3.5 kV; fragmentor, 135 V, and skimmer, 65 V. The detection was performed under the full scan from *m/z* 150 to *m/z* 600.

### Isolation and Identification of Metabolite 1 (M1)

All the protocols involving the use of animals were approved by the Institutional Animal Care and Use Committee of Zhejiang University (Approval ID: SYXK (ZHE) 2005–0072). M1 is one of the metabolites of BYZX in HLMs incubation which also largely exists in the rat urine treated by BYZX. Therefore, M1 was isolated from the rat urine treated by BYZX. 18 male Sprague Dawley rats were fasted for 12 h before intragastric administration with 5 mg/200 g body weight of BYZX (dissolved in 2 mL of 20% β-cyclodextrin), twice a day. The urine samples were collected every 12 h and combined with the addition of acetonitrile (5% total volume), and were kept in the refrigerator at –80°C before use. The collected urine (about 600 mL) was extracted with 2 volumes of ethyl acetate for 3 times, and the supernatants were combined and evaporated to dryness under reduced pressure at 40°C. The residue was redissolved with 15 mL of water/methanol solution (50∶50, v/v) for purification.

M1 isolation was performed on a SinoChrom ODS-BP semi-preparation column (20.0 mm×250 mm, 10 µm, Dalian Elite Analytical Instruments Co., Ltd., Dalian, China) in a P230 LC system equipped with a UV230 detector (Dalian Elite Analytical Instruments Co., Ltd., Dalian, China). The isolation was carried out with two different methanol-water solvent systems at flow rate of 10 mL/min and the absorption peaks were detected at 325 nm. The preliminary elution system of 10 mM ammonium formate in water and methanol (52∶42, v/v) was used to separate out the fraction mainly containing M1, which was then evaporated and extracted for further purification with elution system of 0.1% formic acid in 10 mM ammonium formate solution and methanol (55∶45, v/v). The purified M1 fraction was evaporated and extracted with ethyl acetate to isolate M1 from buffer salt in the elution solvent.

NMR spectra were obtained by using a Bruker AVIII 500 M spectrometer (Fällan- den, Switzerland). Analytes were dissolved in CDCl_3_ solution. The ^1^H, ^13^C, and 2D NMR (^1^H–^1^H COSY, HMBC, and HSQC) analyses were carried out on 12.5 mg/mL solutions of analytes in CDCl_3_ solution.

### Quantitation Analysis by LC-MS/MS

Waters UPLC-TQD system (Waters Acquity, Waters, Milford, MA) was used to establish an LC-MS/MS method for quantitation of BYZX and its major metabolites in HLMs. Chromatographic separation of BYZX and its metabolites were achieved by using an Agilent Eclipse XBD-C_18_ column (2.1 mm×50 mm, 3.5 µm, Agilent, USA) with an infinity in-line filter operating at 35°C. The mobile phase consisted of 0.1% formic acid in water (A) and methanol (B) at a constant flow rate of 0.4 mL/min at a nonlinear gradient program as follow: the initial percentage of mobile B was 2% and raised linearly to 50% in 8.0 min, followed by a further increase to 95% in 0.5 min, maintained for 1.0 min and then rapidly back to 2%. The MS parameters were the same as the aforementioned conditions. Data were acquired by using Masslynx software (version 4.1, Waters) in the multiple reaction monitoring mode for the following transitions: 366.0 *m/z* to 292.9 *m/z* for BYZX, 340.0 *m/z* to 294.9* m/z* for M1, 368.0 *m/z* to 294.9* m/z* for M2, 338.0 *m/z* to 292.9* m/z* for M3, 368.0 *m/z* to 297.0* m/z* for internal standard. Since the amount of M3 was found to be too small to be isolated from both rat urine and HLMs incubations, and the chemical synthesis didn’t achieve successfully, M3 concentrations were given in arbitrary units (A.U.) relative to the peak area ratio of M3 to that of the internal standard in the chromatogram [10].

### Initial Estimation of Metabolism Enzymes

Initial estimation of the main metabolic pathway in HLMs was performed using specific inhibitors, or by altering experimental conditions, to identify CYP and non-CYP metabolic pathway [11]. Three groups of incubation of BYZX with pooled HLMs under different additive processes were carried out in parallel to identify whether the metabolites produced were mediated by CYPs or not. In Group I, the incubation mixture including 0.4 mg/mL pooled HLMs protein, 4 µL of NADP/NADPH solution (0.026 M NADP and 0.012 M NADPH in 1% NaHCO_3_ solution) and 5 mM 1-aminobenzotriazole (ABT, 1 M dissolved in dimethyl sulfoxide, DMSO) were preincubated at 37°C for 30 min, and then 20 µM BYZX was added into the incubation system (n = 2). In Group II, the incubation mixture including 0.4 mg/mL pooled HLMs protein were preincubated at 45°C for 10 min to abolish the activities of the flavin-containing monooxygenases (FMOs), and then 20 µM BYZX was added and precincubated at 37°C for 5 min, and then 4 µL of NADP/NADPH solution was added to initiate the reaction (n = 2). In Group III, the incubation mixture including 0.4 mg/mL pooled HLMs protein and 20 µM BYZX were preincubated at 37°C for 5 min, and then 4 µL of NADP/NADPH solution or 0.1% NaHCO_3_ solution were added to initiate the reaction (n = 2). After 40 min, the reactions were terminated by 1 volume of cold methanol containing internal standard, and the metabolic ratios were compared with that of control group.

### Cytochrome P450 in vitro Reaction Phenotyping for BYZX Metabolism

Further studies on the identification of the human CYPs involved in BYZX metabolism were conducted through three approaches, utilizing chemical inhibitors of CYPs, correlation study, and recombinant human CYP enzymes, respectively.

#### Chemical inhibition analysis in HLMs

The chemical inhibitors for nine CYP isoforms utilized in the metabolic pathway of BYZX to M3 and M2 were α-naphthoflavon (0.02, 0.2, 2 µM) for CYP1A2, pilocarpine (2, 5, 20 µM) for CYP2A6, sertraline (2, 5, 20 µM) for CYP2B6, quercetin (2, 5, 20 µM) for CYP2C8, fluconazole (0.2, 2, 10 µM) for CYP2C9, ticlopidine (2, 5, 20 µM) for CYP2C19, quinidine (0.2, 2, 10 µM) for CYP2D6, sodium diethyldithiocarbamate (10, 20, 40 µM) for CYP2E1 and ketoconazole (0.02, 0.2, 2 µM) for CYP3A4. The concentrations of ketoconazole for CYP3A4 inhibition in the metabolic pathway of M2 to M1 were 0.02, 0.1, 0.2, 2, 10, and 20 µM. The concentrations of BYZX and M2 used were both 20 µM. Control incubations containing all components of the reaction mixtures, including the same amount of DMSO (<1%, v/v) but not the inhibitors, were preformed in parallel. The metabolic activities were calculated as a percentage of control activity, and the concentration that causes 50% inhibition (IC_50_) values were calculated from the fitted curves using the GraphPad Prism computer program (GraphPad Software Inc., San Diego, CA), designed for nonlinear regression analysis. The fitting formulation is as follows: Y = Bottom + (Top – Bottom)/(1+10∧((Log IC_50 –_ X) ×HillSlope)). Where X is the log of dose of concentration; Y is the response, decreasing as X increases; Top and Bottom is the plateaus in same units as Y; log IC_50_ is the same log units as X; HillSlope is the slope factor.

#### Correlation analysis in 10 individual HLMs

The metabolic activities of BYZX and M2 at a concentration of 20 µM were measured in a bank of HLMs from 10 individual donors and then compared with the metabolic activities of CYP-selective probes. Pearson’s product-moment correlation coefficient (*r*) estimated by GraphPad Prism (version 5.0) was used to assess the relationship between formation activities of M1 and M3 and CYP1A2-selective phenacetin O-deethylatioin activity, CYP2A6-selective coumarin 7-hydroxylation activity, CYP2B6-selective efavirenz 8-hydroxylation activity, CYP2C8-selective taxol 6-alpha-hydroxylation activity, CYP2C9-selective tolbutamide 4-methylhydroxylation activity, CYP2C19-selective omeprazole 5-hydroxylation activity, CYP2D6-selective dextromethorphan O-demethylation activity, CYP2E1-selective chlorzoxazone 6-hydroxylation activity, and CYP3A4-selective midazolam 1-hydroxylation activity.

#### Incubation with recombinant human CYPs

Commercial baculovirus-insect cell-expressed human CYPs (0.1 mg/mL) including CYP1A2, 2A6, 2B6, 2C8, 2C9, 2C19, 2D6, 2E1 and 3A4 were incubated with 20 µM BYZX and 3 µM M2 in the aforementioned method respectively. After 40 min 1 volume of ice cold methanol was added to terminate the reaction. The mixture was vortexed for 1 min and centrifuged at 13 000 rpm for 15 min. 7 µL of the supernatant was injected into the LC-MS system for analysis.

Moreover, very small amount of M3 was isolated from pooled incubation samples by HPLC-UV on an ODS column with a gradient solvent system consisting of methanol and 10 mM ammonium formate in water. Then, M3 were incubated in pooled HLMs, recombinant CYPs, and human cytosol to confirm the further metabolism.

#### Metabolism kinetics of BYZX and M2

The kinetics studies were performed using human liver cytosol, HLMs, and recombinant human CYP3A4 and CYP2C8. Before the kinetic study, the incubation time and the enzyme concentration for linear formation of metabolites were optimized. The concentrations of cytosol, HLMs, CYP3A4 and CYP2C8 protein were 0.4, 0.4, 0.04 and 0.1 mg/mL, respectively. The incubation times of HLMs, CYP3A4 and CYP2C8 were 40, 40, 20 and 40 min, respectively. Kinetic parameters were estimated from the fitted curves using the GraphPad Prism computer program (version 5.0, GraphPad Software Inc., San Diego, CA), designed for nonlinear regression analysis. The following equation was applied, assuming a Michaelis–Menten equation: V = *V*_max_× [S]/(*K*_m_+[S]), where V is the rate of reaction, *V*_max_ is the maximum velocity, *K*_m_ is the Michaelis constant (substrate concentration at half of *V*_max_), and [S] is the substrate concentration. Eadie-Hofstee plot was used to confirm allosteric kinetics [12].

### Preliminary Studies on the Hydrogenation of BYZX

#### Incubation with recombinant NADPH-CYP450 oxidoreductase (OR) and cytochrome b_5_ (cyt b_5_)

In order to determine whether OR and cyt b_5_ are the reductases of BYZX, OR and cyt b_5_ were expressed individually and together in our previous method using insect cell expression system, Sf9 cell line [13], [14]. The incubation method was similar to what described above. The production of M2 was determined.

#### Speculation on some other reductases

The reaction rate of BYZX hydrogenation in human liver cytosol had been found to be much higher than that in HLMs, thus it was inferred that the hydrogenation could also happen in liver cytosol. Estimation of the kinetics for BYZX hydrogenation in human liver cytosol was similar to what described above. BYZX in a series of concentration (5.0, 10.0, 20.0, 40.0, 100.0, 150.0, 200.0 µM) were incubated with 0.2 mg/mL of cytosol protein for 20 min at 37°C, and the different formation rates of M2 were assayed for estimating the kinetic parameters.

Some reductase inhibitors and substrate were used to inhibit the hydrogenate reaction of BYZX. 20 µM BYZX was incubated with 100 µM dicoumarol in human liver cytosol and was incubated with 100 µM benzylideneacetone and with 20 µM finasteride in HLMs, respectively. To determine the IC_50_ values, dicoumarol (0.5 to 100 µM) and indometacin (5 to 100 µM) were incubated with 20 µM BYZX and 0.2 mg/mL of protein within 20 min, respectively. The IC_50_ values were calculated from the fitted curves using the GraphPad Prism computer program (GraphPad Software Inc., San Diego, CA), designed for nonlinear regression analysis.

## Results

### Qualitative Analysis of Metabolites in the HLMs by LC-MS

**Figure 1:**
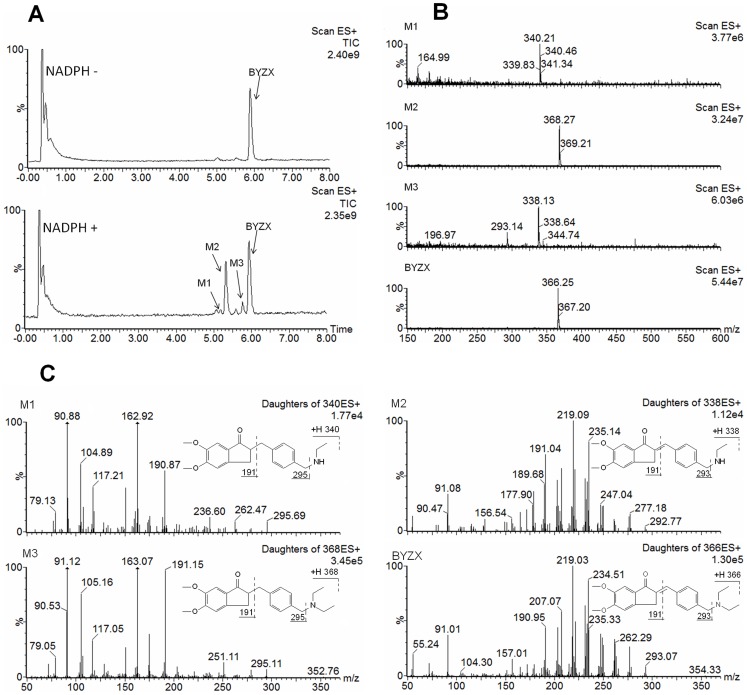
LC-MS chromatograms and mass spectrums of BYZX and its metabolites. A, total ion chromatograms of BYXZ (20 µM) metabolism in pooled HLMs for 20 min without and with NADPH. The peaks at 5.1 and 5.6 min also existed in the incubation matrix. B, MS spectrums of BYZX and its metabolites in the total ion chromatogram. C, MS/MS spectrums and ion fragments analysis of BYZX and its metabolites in the total ion chromatogram.

A full-scan chromatogram of the incubated sample is shown in Fig. 1A. Compared with the matrix control, BYZX showed 3 metabolites, namely M1, M2, and M3. The peaks at 5.1 and 5.6 min also existed in the incubation matrix. Results of accuracy molecular weight and elemental analysis indicated that compared to BYZX (C_23_H_27_O_3_N; observed, 366.2220 and calculated, 366.2269, [M+H]^+^), M1 (C_21_H_25_O_3_N; observed, 340.1907 and calculated, 340.1913, [M+H]^+^) was a [-C_2_H_4_] and hydrogenated metabolite; M2 (C_23_H_29_O_3_N; observed, 368.2220 and calculated, 368.2226, [M+H]^+^) was a hydrogenated metabolite; and M3 (C_21_H_23_O_3_N; observed, 338.1751 and calculated, 338.1756, [M+H]^+^) was a [-C_2_H_4_] metabolite.

Under a collision voltage of 40 eV, BYZX initially produced a fragment ion at *m/z* 293 by loss of the NH-(C_2_H_5_)_2_ group after breakage of the C-N bond. Similar to BYZX, M1 could also lose its NH-C_2_H_5_ group, yielding a fragment ion at *m/z* 295. Similarly, M2 and M3 produced fragment ions at *m/z* 295 and *m/z* 293, respectively. Other fragment ions of M1, M3, and BYZX had similar *m/z* values, which suggested the loss of [-C_2_H_4_] group to yield M1 and *N*-desethylation to yield M3. Mass spectrograms showed a difference in the abundance of the fragment ions between M1 and M3 and between M2 and BYZX (Fig. 1C). This phenomenon indicated that the hydrogenation of BYZX to yield M2 might have broken the conjugate rigid plane structure of BYZX; moreover, M1, which had a structure similar to that of M2, was likely produced by *N*-desethylation of M2.

### Structure Confirmation by NMR

NMR spectra indicated C = C hydrogenation and *N*-desethylation of BYZX incubated in HLMs, which yielded the metabolites M1 and M2. The signals in the region from δ 2.0 to δ 4.0 in the ^1^H-NMR spectrum of M1 were extremely different from those of BYZX (Table 1, position 8, 9, and 10), and the complex splitting indicated multiple coupling of these signals. No hydrogen signal of OH was found in ^1^H-NMR spectrum of M1. The ^13^C-NMR spectrum continued to show the carbon signal of carbonyl in M1, which suggested that the carbonyl group in BYZX did not undergo hydrogenation. ^1^H-^1^H COSY, HMQC, and HMBC spectra were obtained to identify the coupled protons and carbon atoms. All of the signals could be assigned and are presented in Table 1. The multiple splitting of H-8 (δ 2.90) on C-8 (δ 48.84) was because of the 2 protons on C-7 (δ 36.95) and the other 2 on C-9 (δ 31.92); protons (δ 2.58, δ 3.29) on C-7 were subjected to splitting because of the H-8 and themselves, because their chemical equivalence situation was broken as the C-C double bond was broken, which destroyed the formerly planar structure of the molecule. A similar change occurred in the case of the protons (δ 2.69, δ 3.02) on C-9.

**Table 1:** NMR assignments for BYZX, M1 and M2.

Position	δ_H_			δ_C_		
	BYZX	M1	M2	BYZX	M1	M2
1	1.05–1.08, t	1.18–1.21, t	1.02–1.06, t	11.8	11.3	11.6
2	2.52–2.57, q	2.79–2.83, q	2.49–2.55, q	46.9	41.4	46.5
3	3.61, s	3.88, s	3.54, s	57.3	50.1	57.3
4				134.8	129.8	137.7
5	7.60–7.62, d	7.34–7.36, d	7.24–7.26, d	131.1	130.2	138.0
6	7.42–7.44, d	7.20–7.23, d	7.18–7.20, d	129.3	129.4	128.6
7				142.0	140.9	138.0
8	7.36, s	2.51–2.61, dd	2.60–2.70, dd	134.0	37.0	36.9
		3.27–3.31, dd	3.34–3.38, dd			
9		2.87–2.96, m	2.96–3.00, m	134.8	48.8	49.1
10	3.97, s	2.67–2.71, dd	2.75–2.79, dd	32.2	31.9	31.8
		3.00–3.04, dd	3.04–3.10, dd			
11				144.8	148.8	148.9
12	7.60, d	7.17, d	7.17, d	107.2	107.4	107.3
13				155.3	155.7	155.4
14	3.96, s	3.94, s	3.94, s	56.3	56.3	56.1
15	4.00, s	3.90, s	3.92, s	56.1	56.1	56.0
16				149.6	149.5	149.3
17	6.99, s	6.80, s	6.81, s	105.0	104.4	104.3
18				132.0	129.1	129.2
19				193.2	206.1	206.5

The numbers in the first column correspond to those in Fig. 7. All of the signals were assigned with the help of 2D-NMR. The values of **δ**_H_ at position 8∼10 for BYZX are distinct from those for M1 and M2, due to the splitting signals resulting from C = C reduction. The carbonyl signal still remained (Position 19).

According to our assumption, M2 had a structure similar to that of M1, and the former was named a C = C hydrogenated metabolite of BYZX. M2 was synthesized on the basis of this structure [1], and the NMR spectra of M2 resembled those of M1 (Table 1), which confirmed the structure elucidated above. Furthermore, the LC chromatographic retention times of M2 and M1 in the HLMs incubation mixture and the co-spiked sample were found to be the same.

The structure of M3 could be identified directly from its fragment ions as aforementioned LC-MS results. The structures of all the metabolites are presented in Fig. 1C.

### Initial Estimation of the Enzymes Involved in the Metabolism of BYZX

**Figure 2:**
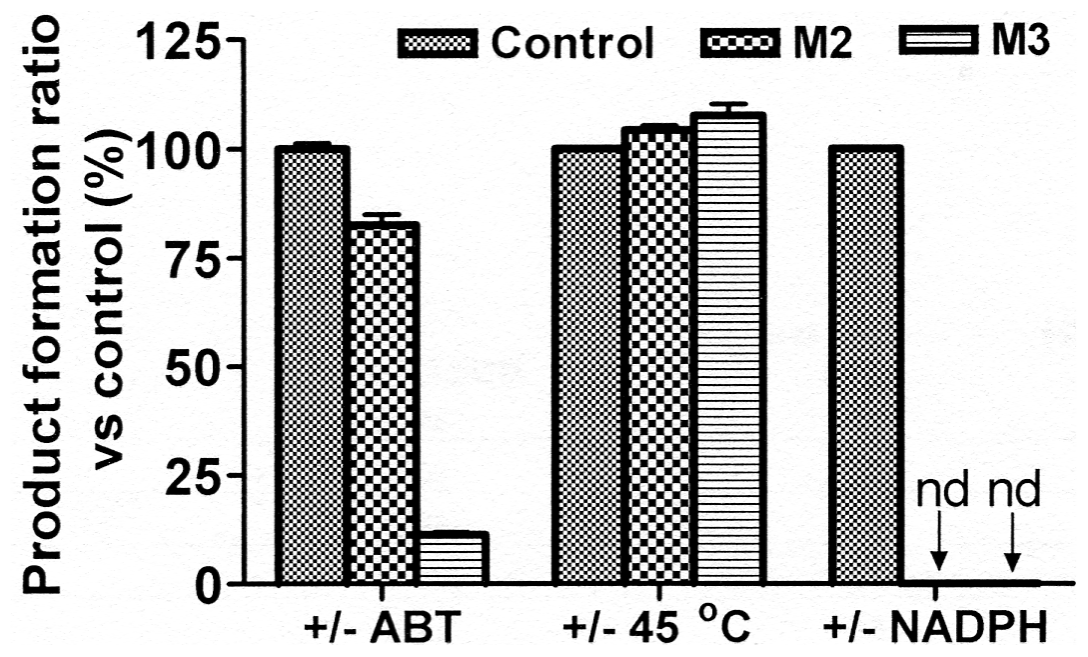
Initial estimation of the main metabolic pathway of BYZX in HLMs. The results are expressed as a percentage of the control activity. nd means no metabolite peak was detected in the LC-MS/MS chromatogram under the chromatographic condition in section 2.5.

The ratios of formation of metabolites in the 3 groups are shown in Fig. 2. NADPH was proven necessary for the metabolism of BYZX as a cofactor to provide hydrogen and electron according to the result of Group III. Studies were performed in Group I and II to determine whether the metabolites were formed by FMOs or CYPs. In Group I, ABT was added as a nonselective inhibitor of CYPs [15]–[17], which extensively suppressed the formation of the desethyl metabolite M3, while in Group II, heating the incubation mixtures at 45°C for 5 min to deactivate the FMOs had no obvious effect on the formation of M3. These results indicated that CYPs were involved in the production of M3. The hydrogenation of BYZX to M2 was not significantly inhibited in Group I and II, which suggested that reductases than CYPs or FMOs may be involved in the hydrogenation in HLMs.

### Cytochrome P450s in vitro Reaction Phenotyping for BYZX Metabolism

**Figure 3:**
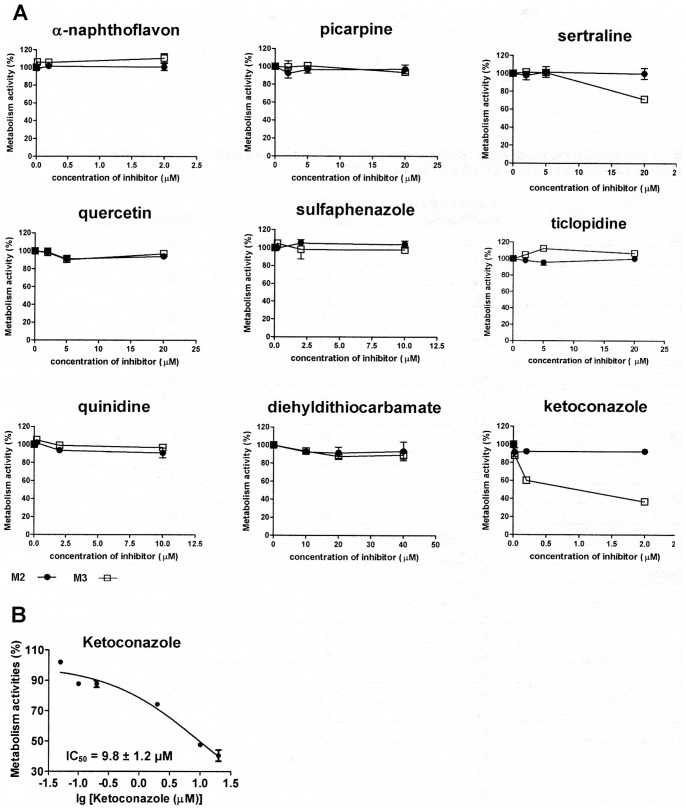
Effects of CYP inhibitors on the formation rates of metabolites in pooled HLMs. A, effects of CYPs typical inhibitors on the formation of M2 and M3 from BYZX. The results were expressed as a percentage of the control activity. B, inhibit effect of ketoconazole on the formation of M1 from M2.

**Figure 4:**
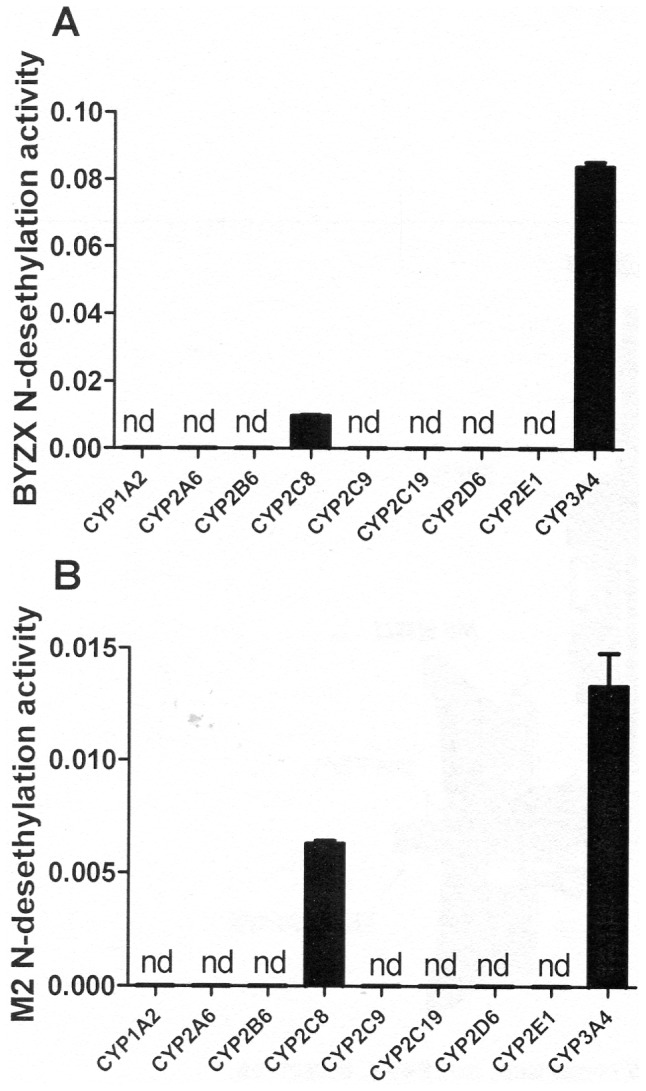
BYZX and M2 N-desethylation metabolic activities in recombinant human CYPs. A, formation of M3 from BYZX. B, formation of M1 from M2. nd means no metabolite peak was detected in the LC-MS/MS chromatogram under the chromatographic condition in section 2.5.

The above results indicated that desethylation was one of the major metabolic pathways mainly mediated by specific CYP isoforms. In the chemical inhibition study, the formation of M3 from BYZX was strongly inhibited by ketoconazole (IC_50_ = 0.6±1.2 µM, Fig. 3A), and the formation of M1 from M2 could be significantly inhibited by ketoconazole (IC_50_ = 9.8±1.2 µM, Fig. 3B). The results of the correlation study (Table 2) indicated that the formation of M3 from BYZX in 10 individual HLMs correlated with the activities of CYP3A4 (r^2^ = 0.82, *P* = 0.0008) and CYP2C8 (r^2^ = 0.62, *P* = 0.021). In addition, the formation of M1 from M2 in 10 individual HLMs correlated with the activities of CYP3A4 (r^2^ = 0.80, *P* = 0.0005) and CYP2C8 (r^2^ = 0.67, *P* = 0.0037). Incubation with different isoforms of human recombinant CYP indicated that M2 was present in all the samples, while M1 and M3 were merely present in the incubations with CYP3A4 and CYP2C8 (Fig. 4). In addition, incubations of M3 with HLMs and human cytosol showed that M3 could be further biotransformed into M1. The above results indicated that the metabolic pathways of desethylation of BYZX and M2 were primarily catalyzed by CYP3A4 and CYP2C8.

**Table 2:** Correlation study in 10 individual HLMs.

CYPs	probe	Concentrationof the probe(µM)	r^2^	
			BYZX	M2
CYP1A2	phenacetin	35	0.061	0.004
CYP2A6	coumarin	2	0.023	0.002
CYP2B6	efavirenz	5	0.064	0.072
CYP2C8	paclitaxel	2	**0.618**	**0.673**
CYP2C9	tolbutamide	73	0.000	0.006
CYP2C19	omeprazole	1.4	0.185	0.090
CYP2D6	dextromethorphan	0.7	0.020	0.286
CYP2E1	chlorzoxazone	70	0.561	0.433
CYP3A4	midazolam	7	**0.821**	**0.798**

### Kinetics of BYZX and M2 Metabolism in Pooled HLMs, Recombinant CYP3A4, and CYP2C8

**Figure 5:**
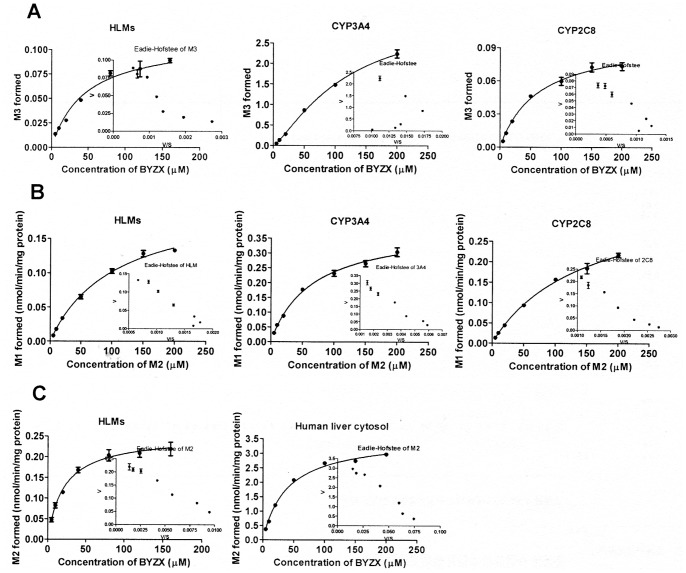
Kinetics of the formation of M3 from BYZX (A) and M1 from M2 (B) in HLMs, CYP3A4 and CYP2C8, and M2 from BYZX (C) in HLMs and human liver cytosol. Each inset shows the Eadie-Hofstee plot of the experimental data.

M2 and M3 were the direct metabolites of BYZX in the HLMs. The kinetics of the formation of M2 and M3 in HLMs showed a classic hyperbolic pattern, and the Michaelis–Menten equation was fitted to calculate the *K*_m_ and *V*_max_ (Fig. 5C and 5A). The *K*_m_ values for biotransformation of BYZX into M2 and M3 were 21.4±1.4 and 46.4±5.9 µM, respectively. The kinetics of BYZX *N*-desethylation (M3 formation) mediated by CYP2C8 fitted the Michaelis–Menten kinetics (r^2^ = 0.9855), which was confirmed by the Eadie–Hofstee plot, and the *K*_m_ value was 62.1±8.2 µM. Compared to the *N*-desethylation mediated by CYP2C8, the *N*-desethylation mediated by CYP3A4 yielded a curve in Eadie–Hofstee plot, which suggested that the metabolism of BYZX mediated by CYP3A4 should typically fit allosteric sigmoidal kinetics [18] (r^2^ = 0.9961), and the *K*_m_ value was 488.2±160.2 µM (Table 3).

**Table 3:** Kinetic parameters of N-des-ethylation of BYZX and M2 in HLMs, CYP3A4 and CYP2C8.

	BYZX				M2		
	HLMs	CYP3A4	CYP2C8		HLMs	CYP3A4	CYP2C8
*K*_m_ (µM)	46.4±5.9	488.2±160.2	62.1±8.2	*K*_m_ (µM)	105.7±10.9	65.2±7.2	143.2±17.7
*V*_max_ (A.U.)	0.111±0.008	3.3±0.4	0.099±0.005	*V*_max_ (nmol/min/mg protein)	0.209±0.010	0.392±0.016	0.370±0.024
*Cl_i_*_nt_ (A.U.)	2.39	6.76	1.59	*Cl_i_*_nt_ (µL/min/mg protein)	1.98	6.01	2.58

M2 could be biotransformed into M1 in pooled HLMs, and the *K*_m_ value was 105.7±10.9 µM. The kinetics of recombinant CYP3A4 and CYP2C8 both fitted the Michaelis–Menten model (Fig. 5B), and the *K*_m_ values were 65.2±7.2 and 143.2±17.7 µM for CYP3A4 and CYP2C8, respectively (Table 3). CYP3A4 exhibited over 2 folds of *Cl*_int_ value compared with that of CYP2C8 (Table 3).

### Preliminary Studies on the Hydrogenation of BYZX

**Figure 6:**
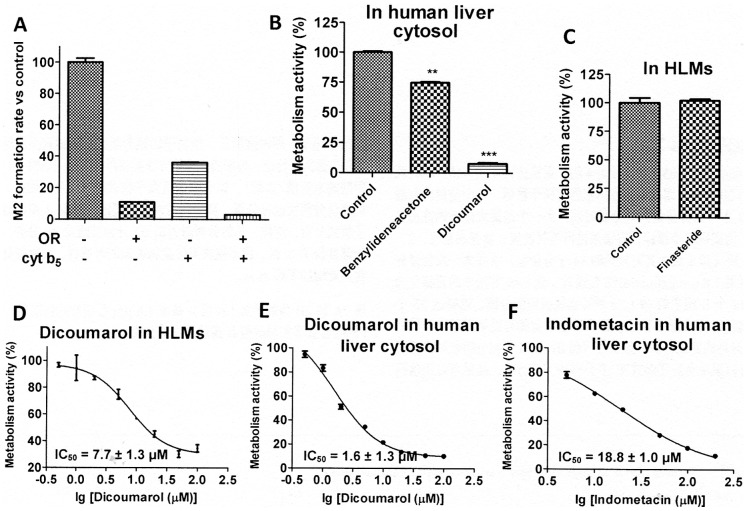
Preliminary studies on the hydrogenation of BYZX. A, effect of recombinant OR and recombinant cyt b5 on the formation of M2 from BYZX. B, effects of benzylideneacetone and dicoumarol on the formation of M2 from BYZX in human liver cytosol. Differences between control and inhibitor group were evaluated using student’s *t*-test. *P*<0.05 was considered to be statistically significant. **, *P*<0.01; ***, *P*<0.001. C, effect of finasteride on the formation of M2 from BYZX in HLMs. D and E, inhibit plots of dicoumarol on the formation of M2 from BYZX in HLMs and human liver cytosol respectively. F, inhibit plot of indometacin on the formation of M2 from BYZX in human liver cytosol.

BYZX could be rapidly metabolized into M2 in the S_9_ fraction of blank Sf9 cells, but the rate of formation of M2 was significantly decreased in the mixture of recombinant OR and cyt b_5_ (Fig. 6A). These results indicated that OR and cyt b_5_ were not involved in the hydrogenation of BYZX. The rate of hydrogenation (*V*_max_) of BYZX in the human liver cytosol was about 15 times higher as that in the HLMs, and the *K*_m_ value in the cytosol was about 2 times (*K*_m = _38.9±2.5 µM, *V*_max = _3.56±0.07 nmol/min/mg protein, *Cl*_int = _91.5 µl/min/mg protein) higher than that in the HLMs (*K*_m = _21.4±1.4 µM, *V*_max = _0.252±0.008 nmol/min/mg protein, *Cl*_int = _11.8 µl/min/mg protein), which suggested that the hydrogenation of BYZX occurred not only in the HLMs but also to a large extent in the human liver cytosol (Fig. 5C). Moreover, the formation of M2 could be extensively suppressed by dicoumarol in both the HLMs (IC_50_ = 7.7±1.2 µM, Fig. 6D) and human liver cytosol (IC_50_ = 1.6±1.3 µM, Fig. 6E) and by indomethacin in the human liver cytosol (IC_50_ = 18.8±1.0 µM, Fig. 6F). In addition, benzylideneacetone (100 µM) significantly suppressed the hydrogenation (*P*<0.05, Fig. 6B). However, finasteride had no inhibitory effect on the hydrogenation of BYZX in the HLMs (Fig. 6C).

## Discussion

BYZX is currently being developed as a potential candidate for therapeutic intervention in neurodegenerative diseases. Although we had previously reported one of the metabolites (M3) of BYZX in the HLMs [9], the result of that study was flawed. Lack of good chromatographic separation and low UV absorption sensitivity may be the 2 reasons for that result. In this study, we found 2 other metabolites in the HLMs by using a highly efficient resolution column in UPLC-MS/MS. In addition, these 3 metabolites were detected in the urine of rats treated with BYZX, but M1 was the only metabolite with a sufficient yield and was suitable for semi-preparative HPLC. Therefore, only M1 was prepared using biological sample.

High-resolution mass spectrum gave the accurate molecular weights of BYZX and its metabolites, and elemental analysis indicated their formulas, but the site of hydrogenation could not be located. Thus, NMR as well as daughter scan using MS/MS were utilized to identify the chemical structures of the metabolites. The signal of the carbonyl group continued to exist in the ^13^C NMR spectra of M1, and the signals of its protons and carbons were successfully assigned using 2D-NMR analyses, both of which confirmed the chemical structure of M1 as an *N*-desethylated and C = C hydrogenation metabolite of BYZX. The fragment ions in the mass spectra of M1 and M2 were identical, which indicate that the only difference between them was *N*-ethyl group. The fragment ions in the mass spectra of BYZX and M3 also indicate that the only difference between them was *N*-ethyl group. The abundance of fragment ions of M1 and M2 was different from that of M3 and BYZX, which presumably resulted from the breakage of the conjugate rigid plane structure by the hydrogenation reaction. The structure of M2 was accurately elucidated by results of the NMR of the synthesized M2, which had a structure identical to that of the metabolite formed in the incubations, which was confirmed by the same retention time, identical MS spectrum, as well as the same metabolic characteristics in vitro.

The results of initial estimation of enzymes involved in the metabolism of BYZX indicated that CYPs were involved in the *N*-desethylation of BYZX. Therefore, 3 approaches, including studies using chemical inhibitors, correlation study, and studies using recombinant human CYPs, were used to identify which CYPs were involved in the *N*-desethylation of BYZX and M2 [19]. The results of recombinant human CYPs and correlation study indicated that CYP3A4 and CYP2C8 were 2 major enzymes catalyzing the *N*-desethylation reaction of BYZX. The difference between the results of studies using chemical inhibitors and those using recombinant human CYPs and correlation studies was because of the substrate overlaid among the CYPs because sertraline used as the specific inhibitor of CYP2B6 was also inhibited CYP3A4 (Fig. 3A) [20], [21]. Besides, the inhibition of CYP2C8 did not have a marked effect on the metabolism, which might be because of a smaller content and weaker activity of CYP2C8 to metabolize BYZX in the HLMs than that of CYP3A4.

To compare the metabolic activities of CYP3A4 and CYP2C8, the kinetics of each enzyme were estimated. Because of no M3 reference for quantitative determination *V*_max_ and *Cl*_int_ of BYZX into M3 can not be accurately determined. However, the estimation *V*_max_ and *Cl*_int_ of BYZX into M3 mediated by CYP3A4 were both significantly higher than that of CYP2C8 which suggested the metabolic elimination of BYZX through desethylation pathway was mainly mediated by CYP3A4 (data not shown). CYP3A4 did not exhibit standard Michaelis–Menten kinetics, but exhibited allosteric kinetics, which might result from homotropic substrate interactions [22], [23], and a sigmoidal characteristic was observed. These results reflect the atypical kinetics widely recognized in CYPs. The central hypothesis of the mechanism underlying BYZX metabolism by CYPs is that simultaneous binding of more than one substrate to an enzyme’s active site results in most atypical kinetics, and these interactions are because of allosteric binding at 2 distinct sites, which is defined as a two-substrate model [22]. This phenomenon is common in CYP3A4 [24], [25] and becomes increasingly recognized in other CYP isoforms [26], [27], and understanding the best-fit model would enable the prediction of in vivo metabolic clearance. The metabolism of M2 into M1 in the HLMs is also mediated by CYP3A4 and CYP2C8. The kinetic parameters are presented in Table 3. Similar to the biotransformation of BYZX into M3 CYP3A4 also exhibited preference compared to CYP2C8 in the biotransformation of M2 into M1.

The results of the structural identifications of the metabolite of BYZX were not according to our expectations because C = C reduction was not as common as C = O reduction in drug metabolism. Interestingly, M2 was found in all human recombinant CYPs incubations, and all of these CYPs contained co-expressed NADPH-CYP, OR, and cyt b_5_; therefore, we speculated that OR and cyt b_5_ might be the related enzymes. The results that the activity of hydrogenation of the blank Sf9 cells was significantly higher than that of the recombinant OR and cyt b5, which indicated that OR and cyt b5 did not catalyze the hydrogenation reaction of BYZX (Fig. 6A).

The results of initial estimation of enzymes involved in the metabolism (Fig. 2) and the chemical structure of M2 indicated that some NADPH-linked C = C reductases might be involved in the hydrogenation of BYZX. Many reductases are known to exist in the cytosol. We found that the hydrogenation activity of BYZX in the human liver cytosol was much higher than that in the HLMs, which indicated that BYZX could be reduced both in the HLMs and in human liver cytosol (Fig. 5C). This finding complicated the identification of the related reductases. The analysis of the α,β-unsaturated ketone structure in BYZX and the C = C reduction reaction indicated a metabolism reaction similar to that mentioned above, and previous studies indicated the role of a kind of NADPH-linked α,β-ketoalkene double bond reductases [28]–[30]. These reductases are widely distributed in the liver cytosol [31]–[33]. Benzylideneacetone, which has a structure similar to a part of BYZX molecule, can be potently hydrogenated by these reductases [28], [31]. Benzylideneacetone can significantly inhibit the formation of M2 (Fig. 6B). Dicoumarol is a well-known reductase inhibitor, including α,β-ketoalkene double bond reductases. In present study, dicoumarol showed a dramatic inhibitory effect on the formation of M2 in the human liver cytosol (Fig. 6B and 6E). According to our results and those reported in previous studies, NADPH-linked α,β-ketoalkene double bond reductases were presumed to be involved in the formation of M2. Unfortunately, NADPH-linked α,β-ketoalkene double bond reductases are only a general designation, and few relevant enzymes were identified and were recombinant expression. LTB4 12-hydroxydehydrogenase/15-oxo-prostaglandin 13-reductase (LTB4 12-HD/PGR), a member of the zinc-independent medium chain dehydrogenase/reductase family have substrate selectivity of LTB4 12-HD/PGR as an alkenal/one oxidoreductase, and this enzyme effectively reduces CS-670, an α,β-ketoalkene similar to BYZX, to a saturated ketone [33]. Dicoumarol and indomethacin were 2 of the inhibitors of LTB4 12-HD/PGR. Dicoumarol and indomethacin inhibited the hydrogenation reaction of BYZX in the human liver cytosol (Fig. 6E and 6F), which suggested that LTB4 12-HD/PGR may be involved in the hydrogenation metabolism of BYZX. However, it should be further identified by the recombinant LTB4 12-HD/PGR enzyme. 5α-Reductase is a well-known enzyme involved in hydrogenation in the microsomes, which converts testosterone to dihydrotestosterone (selective saturation of C = C), which could be significantly suppressed by finasteride [15], [16], [34], [35]. In this study, finasteride showed no inhibitory effect, which indicated that 5α-reductase was not involved in the hydrogenation of BYZX (Fig. 6C). Therefore, further studies are required to identify the reductases involved in the hydrogenation metabolism of BYZX.

Though BYZX can be biotransformed into those metabolites, preclinical pharmacodynamic study had been conducted on animal models to prove a good therapeutic effect on Alzheimer’s disease [1], [3]. Besides, M1 and M2, showed similar pharmacological activity and played as a cholinesterase inhibitor like BYZX did (data not shown). Thus, BYZX has its advantages as a new candidate drug to be further developed.

**Figure 7:**
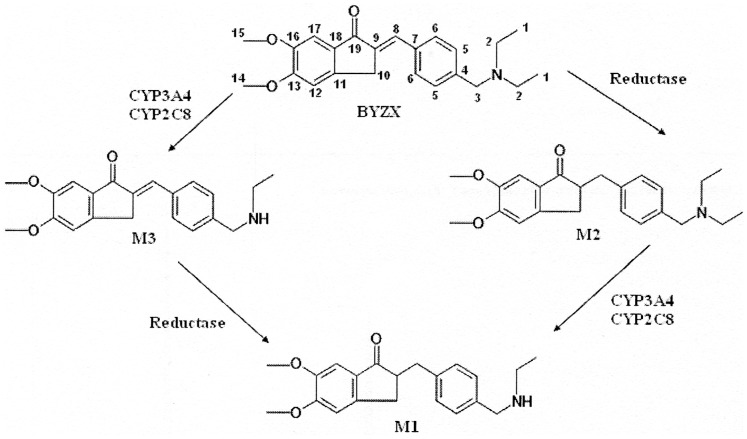
The metabolic pathway of BYZX in HLMs and human liver cytosol.

In conclusion, the major metabolic pathways of BYZX in the human liver microsomes and in the human liver cytosol are hydrogenation and desethylation, which yield a C = C reduction metabolite (M2) and an *N*-desethylation metabolite (M3) both of which can be further biotransformed by *N*-desethylation and C = C hydrogenation, respectively, producing the same metabolite of M1 (Fig. 7). The reductase related to the hydrogenation remains to be identified; however, CYP2C8 and CYP3A4 are shown to be responsible for the desethylation of BYZX and its hydrogenation metabolite (M2).
